# Diagnostic complexity and potentially avoidable invasive procedures before the recognition of pneumoconiosis

**DOI:** 10.3389/fmed.2026.1842645

**Published:** 2026-06-26

**Authors:** Serhat Özgün, Adem Koyuncu, Gülden Sarı, Rabia Ezber, Mücahid Alp Arslan, Ceprail Şimşek

**Affiliations:** Ankara Atatürk Sanatorium Training and Research Hospital, Occupational Diseases Training Clinic, Ankara, Türkiye

**Keywords:** diagnostic complexity, invasive procedures, occupational exposure, occupational lung disease, pneumoconiosis

## Abstract

**Background:**

Pneumoconiosis remains a major occupational health problem worldwide and is frequently initially mistaken for malignancy or infection due to overlapping radiological features. This diagnostic uncertainty may lead to unnecessary invasive procedures, increasing both patient risk and healthcare burden. However, data on how occupational exposure and imaging findings influence diagnostic pathways remain limited.

**Methods:**

In this retrospective single-center study, we evaluated 121 patients with pneumoconiosis who had undergone invasive diagnostic procedures before pneumoconiosis was considered (2021–2025), representing a population with increased diagnostic complexity. We examined occupational exposure, radiological phenotypes, diagnostic decision-making, and procedure-related outcomes. In these patients, in whom pneumoconiosis had not been considered as a preliminary diagnosis, diagnostic concordance was defined as confirmation of the leading preprocedural working diagnosis by invasive findings.

**Results:**

Radiological patterns commonly associated with malignancy or infection were strongly linked to invasive diagnostic procedures. Higher radiological burden was associated with reduced pulmonary function (*p* < 0.001). Diagnostic discordance between initial clinical working diagnosis and invasive findings was observed in a substantial proportion of cases, reflecting challenges in lesion-based diagnostic approaches. The overall complication rate was 10.7%, with pneumothorax being the most frequent adverse event, particularly following percutaneous procedures.

**Conclusion:**

In patients with occupational dust exposure, radiological misinterpretation may contribute to unnecessary invasive diagnostic procedures. Integrating occupational history into early diagnostic evaluation may reduce procedural burden and improve patient safety. These findings highlight the importance of a multidisciplinary and exposure-oriented approach in the management of suspected pneumoconiosis within public health frameworks.

## 1 Introduction

Pneumoconiosis remains a major occupational health problem worldwide, particularly in industries involving prolonged exposure to mineral dusts such as silica, coal, and metal particles. It is categorized within the broader group of occupational interstitial lung diseases (ILDs) and is associated with substantial morbidity and diagnostic complexity (1). Although characteristic imaging patterns often allow a confident diagnosis, a considerable proportion of patients present with atypical radiological features that may mimic malignancy, infection, or inflammatory lung diseases, including hypersensitivity pneumonitis and granulomatous disorders. Such presentations frequently prompt additional imaging, positron emission tomography, or invasive diagnostic procedures, thereby complicating clinical management and increasing procedural exposure as well as contributing to potential overuse of healthcare resources (2). Despite advances in high-resolution imaging and pulmonary function testing, limited data exist regarding how specific computed tomography (CT) phenotypes influence the selection of invasive diagnostic strategies in occupational cohorts. In particular, the procedural implications of mass-like lesions, progressive massive fibrosis (PMF), consolidation patterns, and mediastinal lymphadenopathy remain insufficiently characterised in routine practice.

Accurate diagnosis of occupational lung diseases requires a multidisciplinary framework integrating clinical, radiological, pathological, and exposure data (3). In routine clinical practice, invasive procedures are frequently performed before pneumoconiosis is considered, particularly when occupational exposure is not recognised at initial presentation and radiologically suspicious lesions are instead attributed to malignancy, infection or interstitial lung disease. However, the determinants of diagnostic discordance between initial working diagnoses and final diagnoses among patients undergoing invasive evaluation have not been systematically evaluated. Understanding these discordances is clinically relevant, as they may reflect uncertainty in radiological interpretation, overlap with malignant or infectious processes, or variability in exposure assessment, and may also contribute to potentially avoidable invasive diagnostic procedures when occupational etiology is not adequately considered.

Furthermore, procedure-related complication profiles in pneumoconiosis populations—particularly when stratified according to radiological phenotype and occupational exposure characteristics—remain poorly defined (4). Given that many patients undergo transthoracic needle biopsy, bronchoscopic procedures, or surgical interventions based on imaging suspicion, clarifying phenotype-driven procedural risk has direct implications for patient safety and for reducing unnecessary procedural burden at a population level.

Accordingly, the present study aimed to comprehensively evaluate radiological phenotypes, pulmonary function parameters, occupational exposure characteristics, procedural utilisation patterns, diagnostic concordance, and procedure-related complications in a cohort of pneumoconiosis patients undergoing invasive diagnostic evaluation prior to the recognition of their occupational disease. By examining how occupational exposure and imaging findings shape diagnostic pathways, this study also aims to highlight opportunities to reduce unnecessary invasive procedures and improve decision-making within an occupational and public health context. By identifying phenotype- and exposure-related determinants of diagnostic pathways, we sought to provide clinically relevant evidence to support more structured and safer decision-making in occupational lung disease practice.

## 2 Materials and methods

### 2.1 Study design and setting

This retrospective observational study was conducted at a tertiary referral center specializing in occupational lung diseases. The study included patients in whom pneumoconiosis had not been considered at the centers where they were first evaluated. At these centers, the diagnostic work-up for nodular, mass-like, or radiologically suspicious pulmonary lesions had prioritized alternative diagnoses requiring invasive procedures for pathological or microbiological confirmation, such as malignancy, tuberculosis, interstitial lung disease, or atypical infection. The vast majority of these patients were subsequently referred to our center, while a small proportion presented on their own initiative because of persistent symptoms or disease progression. At our center, all patients underwent comprehensive occupational assessment, and the final diagnosis and occupational attribution of pneumoconiosis were established by the Occupational Diseases Clinic through multidisciplinary evaluation incorporating exposure history, imaging findings, and clinical assessment.

The study protocol was approved by the Ethics Committee of Ankara Ataturk Sanatorium Training and Research Hospital (approval number: 2024-BÇEK/419; 10/12/2025). The study was conducted in accordance with the Declaration of Helsinki and relevant national regulations. Due to the retrospective design and use of anonymized clinical data, the requirement for informed consent was waived by the ethics committee.

### 2.2 Study population

Among 550 patients diagnosed with pneumoconiosis at our clinic between January 2021 and December 2025, 121 (approximately 22%) patients who had undergone at least one invasive diagnostic procedure before pneumoconiosis was considered, and had complete imaging and pulmonary function data were included in the final analysis. Accordingly, the present cohort represents a selected subset of pneumoconiosis patients with radiologically complex or atypical presentations that had prompted invasive diagnostic procedures before the occupational etiology was recognized. Patients undergoing invasive procedures in whom pneumoconiosis was excluded were not systematically recorded, and therefore were not included in the present analysis.

Patients were excluded if they:

1. did not undergo invasive diagnostic procedures,
2. had incomplete thoracic imaging,
3. had missing pulmonary function data,
4. or had duplicate medical records.

The patient selection process is summarized in Supplementary Figure 1.

Following referral, all included patients had a final diagnosis of pneumoconiosis established by the Occupational Diseases Clinic based on compatible chest imaging findings (chest radiography and/or thoracic computed tomography) and documented occupational exposure history, evaluated within a multidisciplinary framework.

### 2.3 Radiological assessment

Thoracic computed tomography (CT) images were reviewed to characterize radiological phenotypes, including:

1. Progressive massive fibrosis (PMF)
2. Mass-like lesions
3. Consolidation
4. Cavitation
5. Mediastinal lymphadenopathy
6. Pleural abnormalities
7. Lesion localization

These CT-based categories were defined to reflect clinically relevant imaging patterns that may influence differential diagnosis and the selection of invasive diagnostic procedures in routine practice.

Progressive massive fibrosis was considered a marker of advanced (complicated) pneumoconiosis in accordance with established radiological definitions.

Radiological profusion was assessed using chest radiography according to the International Labour Organization (ILO) classification system. A profusion category of ≥3 was operationally defined as representing high radiological burden in the present study, consistent with more advanced disease categories within the ILO framework, although this threshold is not universally standardized.

No formal CT-based scoring system (e.g., ICOERD) was applied. Instead, CT findings were interpreted descriptively to capture clinically meaningful radiological patterns relevant to diagnostic decision-making.

### 2.4 Diagnostic procedures

Diagnostic interventions included:

1. Positron emission tomography–computed tomography (PET-CT)
2. Fiberoptic bronchoscopy with transbronchial biopsy (FOB/TBB)
3. Endobronchial ultrasound–guided sampling (EBUS)
4. Transthoracic needle biopsy (TTNB)
5. Video-assisted thoracoscopic surgery (VATS)

Eligibility required that patients had undergone at least one invasive procedure prior to referral, before pneumoconiosis was diagnosed. Each invasive procedure corresponded to a single lesion-specific diagnostic outcome and was directed at the lesion that had prompted the procedure.

Procedural selection was based on radiological phenotype, lesion accessibility, and routine clinical judgment at the time of evaluation. No standardized procedural algorithm was applied, reflecting routine clinical practice.

Procedure-related complications were defined as clinically documented adverse events occurring within 30 days of the invasive intervention and requiring medical evaluation or treatment. Recorded complications included pneumothorax and clinically significant bleeding.

### 2.5 Clinical variables

Demographic characteristics, smoking status, cumulative smoking exposure (pack-years), occupational category, exposure duration, pulmonary function parameters (FVC, FEV_1_, FEV_1_/FVC), comorbidities, and initial working diagnoses prior to invasive procedures were extracted from electronic medical records.

Spirometry was performed using the Zan 100 flow-sensitive spirometry device (ZAN Messgeräte GmbH, Oberthulba, Germany). Calibration was performed daily, and measurements met American Thoracic Society/European Respiratory Society acceptability and reproducibility standards. Pulmonary function parameters were expressed as percentages of predicted values.

Occupational exposure history was obtained from electronic medical records and standardized occupational disease evaluation forms routinely used in the clinic. Exposure assessment was based on detailed occupational history, including job title, industry type, duration of exposure, and known exposure to specific dust types (e.g., silica, coal, metal dust, mixed dust). All exposure data were evaluated by experienced occupational disease specialists within a multidisciplinary framework. Exposure classification was based on clinical judgment and documented occupational history rather than quantitative exposure measurements.

Radiological findings were interpreted in conjunction with formal radiology reports as part of routine multidisciplinary clinical evaluation.

### 2.6 Diagnostic concordance

Diagnostic concordance was defined as agreement between the leading preprocedural working diagnosis that prompted invasive evaluation (e.g., malignancy, infection, tuberculosis, hypersensitivity pneumonitis) and the diagnosis established from the invasive diagnostic procedure (i.e., histopathological or microbiological findings of the sampled lesion).

In this framework, concordance reflected whether the clinician’s initial working diagnosis for the targeted lesion was confirmed by the invasive procedure. Each invasive procedure yielded a single lesion-specific diagnostic outcome. Therefore, if the suspected condition was confirmed, the case was classified as concordant; if not, it was classified as discordant.

Pneumoconiosis was diagnosed only after referral, through multidisciplinary evaluation based on occupational exposure history and imaging findings and was not included in the lesion-specific diagnostic endpoint used for concordance assessment.

### 2.7 Statistical analysis

Statistical analyses were performed using SPSS Statistics version 23 (IBM Corp., Armonk, NY, United States).

Continuous variables were summarized as mean ± standard deviation or median (interquartile range), as appropriate. Normality was assessed using the Shapiro–Wilk test.

Group comparisons were performed using:

1. Student’s *t*-test or Mann–Whitney U test for continuous variables
2. Chi-square or Fisher’s exact test for categorical variables

Correlations were assessed using Spearman’s rank correlation coefficient.

Ordinal logistic regression was used to evaluate predictors of profusion severity after verification of the proportional odds assumption. Multicollinearity was assessed using variance inflation factors (VIF), with values <5 considered acceptable.

Logistic regression models evaluating predictors of diagnostic discordance were considered exploratory due to limited event counts relative to candidate predictors.

All statistical tests were two-sided, and a *p*-value < 0.05 was considered statistically significant.

## 3 Results

### 3.1 Patient characteristics

A total of 121 patients with radiologically confirmed pneumoconiosis were included. The mean age was 55.1 ± 12.2 years, and the mean occupational exposure duration was 20.6 ± 10.5 years. Mean FVC was 81.2 ± 24.2% predicted, and median FEV_1_ was 78% (IQR 54–92). Median cumulative smoking exposure was 20 pack-years (IQR 15–32); 19.0% were never smokers, 42.1% ex-smokers, and 38.8% current smokers.

The most frequent occupational groups were miners (24.8%), welders (20.7%), foundry workers (13.2%), dental technicians (11.6%), and stoneworkers (9.1%). With respect to the specific type of pneumoconiosis, silicosis was the most frequent diagnosis (*n* = 98, 81.0%), followed by welders’ pneumoconiosis (*n* = 22, 18.2%) and a single case of asbestosis (*n* = 1, 0.8%), reflecting the predominance of silica exposure across the included occupational groups.

Radiologically, profusion category distribution was 20.7% (category 1), 62.0% (category 2), and 17.4% (category 3). PMF was present in 56.2%. Pulmonary mass-like lesions were observed in 58.7%, consolidation in 53.7%, lymphadenopathy in 81.8%, cavitation in 3.3%, pleural effusion in 9.1%, and atypical lesion localization in 9.1%.

Positron emission tomography–computed tomography was performed in 74.4% of patients. FOB/TBB, EBUS, TTNB, and VATS were performed in 53.7%, 38.8%, 19.0%, and 15.7% of patients, respectively. Multiple invasive procedures were undertaken in 24.8%. Overall complication rate was 10.7% (Table 1).

**TABLE 1:** Baseline demographic, clinical, radiological, and functional characteristics of the study population, stratified by occupational group.

Variable	Miner (*n* = 30)	Welding (*n* = 25)	Foundry (*n* = 16)	Dental tech. (*n* = 14)	Stonework (*n* = 11)	Other (*n* = 25)
Demographic and clinical characteristics						
Age, years, median (Q1–Q3)	66 (60–75)	49 (42–56)	48 (40–64)	54 (46–61)	56 (54–64)	47 (42–59)
Exposure duration, years, median (Q1–Q3)	20 (16–27)	25 (16–32)	17 (13–20)	27 (10–30)	29 (9–37)	11 (7–25)
Pack-years, median (Q1–Q3)	20 (11–42)	25 (15–32)	25 (15–30)	23 (9–46)	25 (12–44)	20 (10–30)
Smoking status, n (%)						
Never smoker	6 (20.0)	1 (4.0)	4 (25.0)	3 (21.4)	3 (27.3)	6 (24.0)
Ex-smoker	15 (50.0)	12 (48.0)	3 (18.8)	4 (28.6)	6 (54.5)	11 (44.0)
Current smoker	9 (30.0)	12 (48.0)	9 (56.2)	7 (50.0)	2 (18.2)	8 (32.0)
Pulmonary function						
FVC (% pred), median (Q1–Q3)	76 (63–99)	90 (78–109)	82 (64–105)	64 (49–73)	76 (64–92)	81 (57–91)
FEV_1_ (% pred), median (Q1–Q3)	66 (50–93)	86 (74–100)	79 (53–95)	51 (41–76)	72 (61–82)	66 (49–85)
FEV_1_/FVC (%), median (Q1–Q3)	70 (65–78)	78 (73–82)	74 (67–77)	72 (64–76)	75 (64–79)	73 (58–80)
Radiological findings, n (%)						
Profusion category 3	8 (26.7)	3 (12.0)	0 (0.0)	4 (28.6)	2 (18.2)	4 (16.0)
PMF	18 (60.0)	3 (12.0)	6 (37.5)	9 (64.3)	10 (90.9)	22 (88.0)
Mass-like lesion	19 (63.3)	5 (20.0)	7 (43.8)	9 (64.3)	10 (90.9)	21 (84.0)
Consolidation	13 (43.3)	19 (76.0)	9 (56.2)	10 (71.4)	3 (27.3)	11 (44.0)
Lymphadenopathy	24 (80.0)	15 (60.0)	14 (87.5)	14 (100.0)	10 (90.9)	22 (88.0)
Cavitation	3 (10.0)	0 (0.0)	0 (0.0)	0 (0.0)	0 (0.0)	1 (4.0)
Pleural effusion	5 (16.7)	0 (0.0)	0 (0.0)	0 (0.0)	3 (27.3)	3 (12.0)
Atypical lesion localization	2 (6.7)	1 (4.0)	0 (0.0)	2 (14.3)	3 (27.3)	3 (12.0)
Diagnostic procedures, n (%)						
PET-CT	26 (86.7)	9 (36.0)	10 (62.5)	11 (78.6)	11 (100.0)	23 (92.0)
FOB/TBB	13 (43.3)	23 (92.0)	9 (56.2)	3 (21.4)	5 (45.5)	12 (48.0)
EBUS	16 (53.3)	2 (8.0)	7 (43.8)	10 (71.4)	4 (36.4)	8 (32.0)
TTNB	9 (30.0)	1 (4.0)	3 (18.8)	2 (14.3)	4 (36.4)	4 (16.0)
VATS	4 (13.3)	2 (8.0)	0 (0.0)	1 (7.1)	3 (27.3)	9 (36.0)
Multiple invasive procedures	12 (40.0)	3 (12.0)	3 (18.8)	2 (14.3)	4 (36.4)	6 (24.0)
Procedure-related outcomes, n (%)						
Any complication	5 (16.7)	3 (12.0)	1 (6.2)	1 (7.1)	0 (0.0)	3 (12.0)
Pneumothorax	4 (13.3)	3 (12.0)	0 (0.0)	0 (0.0)	0 (0.0)	2 (8.0)
Bleeding	1 (3.3)	0 (0.0)	1 (6.2)	1 (7.1)	0 (0.0)	1 (4.0)
Hospital admission	5 (16.7)	3 (12.0)	1 (6.2)	1 (7.1)	0 (0.0)	3 (12.0)

Data are presented as median (Q1–Q3) or *n* (%). The “Other” category comprises sandblasting (*n* = 7), ceramics manufacturing (*n* = 5), tunnel construction (*n* = 3), marble work (*n* = 3), glass work (*n* = 3), construction (*n* = 2), engineered stone (*n* = 1), and asbestos (*n* = 1) workers. FVC, forced vital capacity; FEV_1_, forced expiratory volume in 1 second; PMF, progressive massive fibrosis; PET-CT, positron emission tomography–computed tomography; FOB/TBB, fiberoptic bronchoscopy with transbronchial biopsy; EBUS, endobronchial ultrasound; TTNB, transthoracic needle biopsy; VATS, video-assisted thoracoscopic surgery.

### 3.2 Radiological features and diagnostic procedures

Radiological phenotypes were strongly associated with diagnostic procedure selection (Table 2). The presence of a pulmonary mass significantly increased TTNB utilization (29.6% vs. 4.0%, *p* < 0.001) and decreased FOB/TBB use (38.0% vs. 76.0%, *p* < 0.001). A similar pattern was observed in patients with PMF, who underwent TTNB more frequently (29.4% vs. 5.7%, *p* = 0.001) and FOB/TBB less frequently (36.8% vs. 75.5%, *p* < 0.001). Consolidation was associated with higher FOB/TBB use (66.2% vs. 39.3%, *p* = 0.003) and lower TTNB use (10.8% vs. 28.6%, *p* = 0.013). Lymphadenopathy was associated with reduced FOB/TBB use (49.5% vs. 72.7%, *p* = 0.048) and increased EBUS use (43.4% vs. 18.2%, *p* = 0.028). Pleural effusion and cavitary lesions were not significantly associated with specific procedural selection.

**TABLE 2:** Association between radiological features and diagnostic procedure selection in patients with pneumoconiosis (*n* = 121).

Radiological feature	Diagnostic procedure	Feature (+) *n*/*N* (%)	Feature (−) *n*/*N* (%)	*P*-value
PMF	FOB/TBB	25/68 (36.8%)	40/53 (75.5%)	<0.001*
	TTNB	20/68 (29.4%)	3/53 (5.7%)	0.001*
	EBUS	27/68 (39.7%)	20/53 (37.7%)	0.825
	VATS	14/68 (20.6%)	5/53 (9.4%)	0.094
Pulmonary mass	FOB/TBB	27/71 (38.0%)	38/50 (76.0%)	<0.001*
	TTNB	21/71 (29.6%)	2/50 (4.0%)	<0.001*
	EBUS	27/71 (38.0%)	20/50 (40.0%)	0.827
	VATS	15/71 (21.1%)	4/50 (8.0%)	0.051
Atypical lesion location	FOB/TBB	4/11 (36.4%)	61/110 (55.5%)	0.226
	TTNB	5/11 (45.5%)	18/110 (16.4%)	0.034*
	EBUS	4/11 (36.4%)	43/110 (39.1%)	1.000
	VATS	4/11 (36.4%)	15/110 (13.6%)	0.070
Pleural effusion	FOB/TBB	3/11 (27.3%)	62/110 (56.4%)	0.065
	TTNB	4/11 (36.4%)	19/110 (17.3%)	0.218
	EBUS	4/11 (36.4%)	43/110 (39.1%)	1.000
	VATS	4/11 (36.4%)	15/110 (13.6%)	0.070
Consolidation	FOB/TBB	43/65 (66.2%)	22/56 (39.3%)	0.003*
	TTNB	7/65 (10.8%)	16/56 (28.6%)	0.013*
	EBUS	20/65 (30.8%)	27/56 (48.2%)	0.050
	VATS	10/65 (15.4%)	9/56 (16.1%)	0.918
Cavity	FOB/TBB	3/4 (75.0%)	62/117 (53.0%)	0.623
	TTNB	1/4 (25.0%)	22/117 (18.8%)	0.575
	EBUS	1/4 (25.0%)	46/117 (39.3%)	1.000
	VATS	1/4 (25.0%)	18/117 (15.4%)	0.500
LAP	FOB/TBB	49/99 (49.5%)	16/22 (72.7%)	0.048*
	TTNB	21/99 (21.2%)	2/22 (9.1%)	0.242
	EBUS	43/99 (43.4%)	4/22 (18.2%)	0.028*
	VATS	16/99 (16.2%)	3/22 (13.6%)	1.000

*Pearson’s chi-squared test. PMF, progressive massive fibrosis; FOB/TBB, fiberoptic bronchoscopy with transbronchial biopsy; EBUS, endobronchial ultrasound; TTNB, transthoracic needle biopsy; HP, hypersensitivity pneumonitis; LAP, lymphadenopathy, VATS, video-assisted thoracoscopic surgery.

Occupational subgroup analyses revealed distinct patterns (Supplementary Table 1). Welders more frequently underwent FOB/TBB (92.0% vs. 43.8%, *p* < 0.001) and less frequently TTNB (4.0% vs. 22.9%, *p* = 0.042) and EBUS (8.0% vs. 46.9%, *p* < 0.001). In contrast, dental technicians underwent EBUS more frequently (71.4% vs. 34.6%, *p* = 0.008) and FOB/TBB less frequently (21.4% vs. 57.9%, *p* = 0.010).

Further analysis demonstrated that welders exhibited distinct clinical–radiological profiles (Supplementary Table 2). Compared with non-welders, welders had a higher frequency of an initial working diagnosis of hypersensitivity pneumonitis (44.0% vs. 5.2%, *p* < 0.001), lower prevalence of PMF (12.0% vs. 67.7%, *p* < 0.001) and mass-like lesions (20.0% vs. 68.8%, *p* < 0.001), and higher prevalence of consolidation (76.0% vs. 47.9%, *p* = 0.012).

### 3.3 Functional correlates of radiological severity

Radiological severity was associated with impaired pulmonary function. Patients with PMF were older (58.3 ± 11.8 vs. 51.1 ± 11.7 years, *p* = 0.001) and had significantly lower FVC (71.0 ± 22.4% vs. 94.3 ± 20.0%, *p* < 0.001), whereas exposure duration did not differ.

Higher profusion burden (≥3) was associated with lower FVC (68.0 ± 26.4% vs. 84.0 ± 22.9%, *p* = 0.005) and lower FEV_1_ (*p* = 0.004). Smoking burden and FEV_1_/FVC ratio did not differ significantly.

Spearman analysis demonstrated an inverse correlation between profusion score and FVC (ρ = −0.258, *p* = 0.005).

**FIGURE 1:**
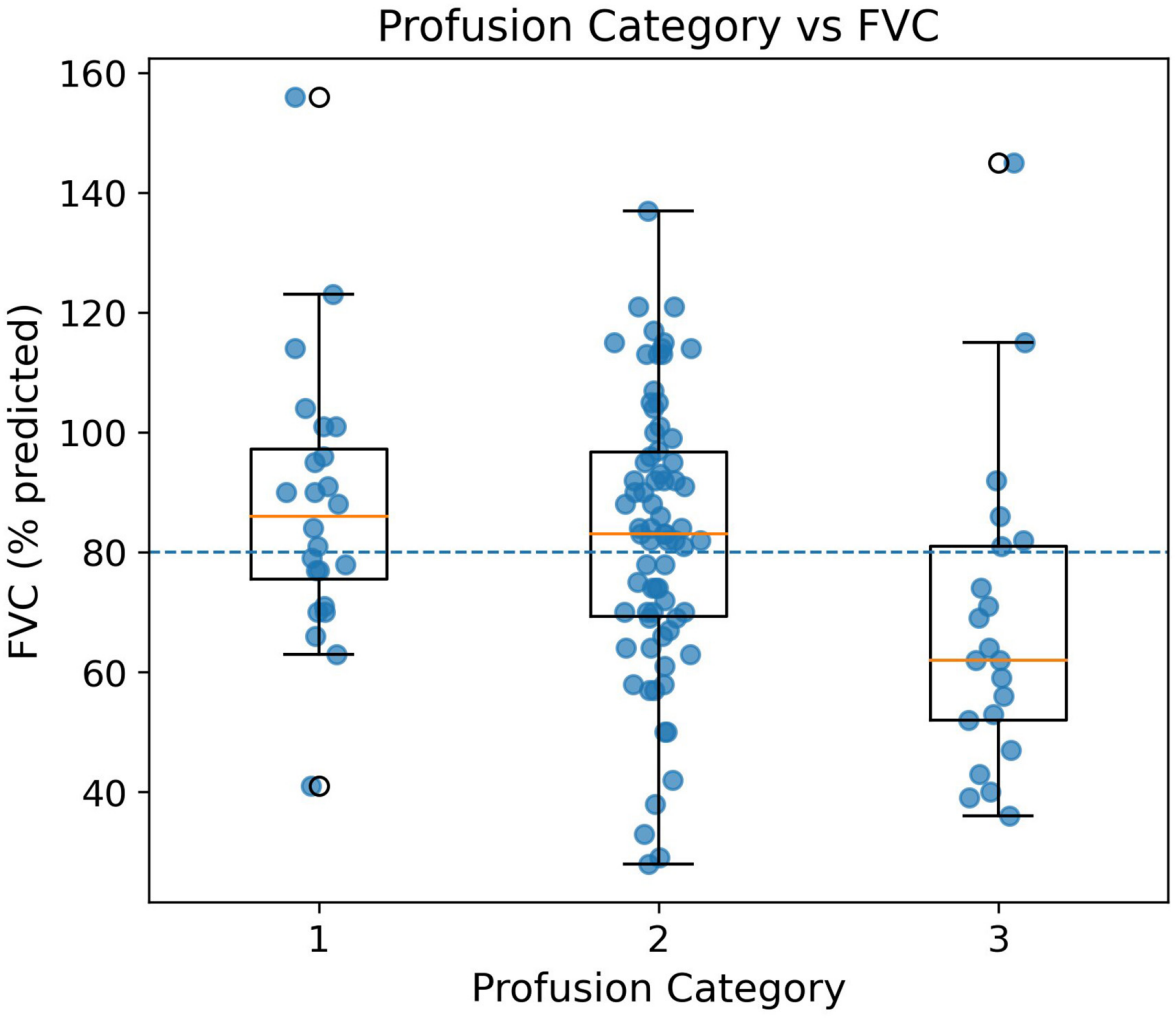
Decline in forced vital capacity (FVC) across increasing radiological profusion categories. This trend reflects a progressive restrictive impairment associated with increasing radiological burden.

In ordinal logistic regression, higher FVC was independently associated with lower profusion categories (OR 0.98 per 1% increase, *p* = 0.008), while age and exposure duration were not significant predictors (Figure 1 and Table 3).

**TABLE 3:** Association between radiological severity and pulmonary function parameters.

Radiological variable	Comparison group	*n*	Pulmonary function/clinical variable [mean ± SD or median (IQR)]	*P*-value
PMF status	PMF+ vs PMF−	68 vs. 53	Age (years): 58.3 ± 11.8 vs. 51.1 ± 11.7	0.001*
			Exposure duration (years): 21.5 ± 11.6 vs. 19.5 ± 9.0	0.288
			FVC (% pred): 71.0 ± 22.4 vs. 94.3 ± 20.0	<0.001*
Profusion category	≥3 vs. <3	21 vs. 98	FVC (% pred): 68.0 ± 26.4 vs. 84.0 ± 22.9	0.005*
			FEV_1_ (% pred): 54 (43–73) vs. 80 (60–95)	0.004**
			Pack-years: 23 (13–37) vs. 20 (15–30)	0.853
			FEV_1_/FVC (%): 73 (67–77) vs. 74 (66–80)	0.762
Correlation analysis	Profusion score ↔ FVC	119	Spearman ρ = −0.26	0.005***
Ordinal logistic regression	Dependent variable: profusion score	121	FVC: B = −0.02 (OR = 0.98 per 1% increase)	0.008****
			Age	>0.05
			Exposure duration	>0.05

*t-test. **Mann–Whitney U test. ***Spearman correlation. ****Ordinal logistic regression. PMF, progressive massive fibrosis; FVC, forced vital capacity; FEV_1_, forced expiratory volume in one second.

### 3.4 Determinants of initial diagnostic impressions

Initial working diagnoses varied according to age, pulmonary function, and occupational exposure (Supplementary Table 3). Infectious suspicion was associated with lower FVC (54.0% ± 23.0% vs. 82.2% ± 23.8%, *p* = 0.022). Patients suspected of malignancy were older (58.7% ± 12.8 vs. 51.6 ± 10.6 years, *p* = 0.001). Patients suspected of hypersensitivity pneumonitis were younger (48.7 ± 9.1 vs. 56.1 ± 12.4 years, *p* = 0.023) and had higher FVC (93.3% ± 24.0% vs. 79.3% ± 23.8%, *p* = 0.031). Welding occupation was strongly associated with hypersensitivity pneumonitis working diagnosis (OR ≈ 14.3, *p* < 0.001). Sarcoidosis suspicion was associated with higher FVC (94.1% ± 21.5% vs. 79.4% ± 24.1%, *p* = 0.027).

### 3.5 Determinants of diagnostic concordance

Overall diagnostic concordance between the leading preprocedural working diagnosis and the diagnosis established through invasive evaluation was observed in 17.4% of patients, whereas 82.6% demonstrated diagnostic discordance. Diagnostic concordance was defined as confirmation of the clinician’s initial working diagnosis that prompted the invasive procedure, based on the lesion-specific diagnostic result (histopathological or microbiological findings). Cases in which the initial clinical working diagnosis was not confirmed by invasive evaluation were classified as discordant. This definition reflects agreement at the level of the targeted lesion rather than the full multidisciplinary diagnosis, as pneumoconiosis was established separately based on imaging findings and occupational exposure assessment.

In univariable analyses, the presence of at least one comorbidity was associated with higher diagnostic concordance (76.2% vs. 51.0%, *p* = 0.035). No significant differences were observed between concordant and discordant groups with respect to age, exposure duration, pulmonary function parameters, presence of PMF, or radiological profusion severity (all *p* > 0.05) (Table 4).

**TABLE 4:** Diagnostic concordance and predictors of diagnostic discordance.

Variable	Diagnostic concordance (*n* = 21)	Diagnostic discordance (*n* = 100)	*P*-value	Effect size
Any comorbidity present, *n* (%)	16 (76.2%)	51 (51.0%)	0.035*	OR = **0.33** (95% CI: 0.11–0.96)
No comorbidity, *n* (%)	5 (23.8%)	49 (49.0%)	Reference	–
Age, years (mean ± SD)	57.3 ± 11.4	54.7 ± 12.4	0.376	Cohen’s *d* = 0.22
Exposure duration, years (mean ± SD)	21.3 ± 10.7	20.5 ± 10.6	0.749	Cohen’s *d* = 0.08
FVC (% predicted, mean ± SD)	75.0 ± 25.3	82.5 ± 23.9	0.194	Cohen’s *d* = −0.31
PMF present, *n* (%)	13 (61.9%)	55 (55.0%)	0.562	OR = 0.75 (95% CI: 0.29–1.97)
Profusion ≥3, *n* (%)	6 (28.6%)	15 (15.0%)	0.135	OR = 0.44 (95% CI: 0.15–1.32)

*Pearson’s chi-squared test. FVC, forced vital capacity; PMF, progressive massive fibrosis. Categorical variables were compared using Pearson χ^2^ or Fisher’s exact test; continuous variables using Student’s *t*-test. Effect sizes are presented as odds ratios (OR) or Cohen’s *d*. Multivariable logistic regression analyses were considered exploratory because of sparse event counts in several subgroups. Bold values indicate statistical significance (*P* < 0.05).

In exploratory multivariable logistic regression analysis, absence of comorbidity remained independently associated with a lower likelihood of diagnostic concordance (OR 0.33, 95% CI 0.11–0.96, *p* = 0.041). No other clinical, functional, or radiological variables were identified as independent predictors.

### 3.6 Procedure-related complications and safety outcomes

Procedure-related complications were observed in 13 patients (10.7%). Pneumothorax occurred in nine patients (7.4%), and clinically significant bleeding in four patients (3.3%). No procedure-related mortality was recorded.

Transthoracic needle biopsy was strongly associated with pneumothorax (26.1% vs. 3.1%, *p* < 0.001; OR 11.18, 95% CI 2.55–49.04). No significant associations were identified between pneumothorax and FOB/TBB, EBUS, or VATS. Bleeding events were not significantly associated with any specific procedure, although a borderline increase was observed following VATS (10.5% vs. 2.0%, *p* = 0.055).

Patients undergoing multiple invasive procedures exhibited a numerically higher complication rate compared with those undergoing a single procedure (20.0% vs. 7.7%, *p* = 0.059).

Mining occupation was associated with a higher likelihood of undergoing multiple procedures (OR 2.70, 95% CI 1.11–6.61, *p* = 0.026), but not with overall complication risk (Table 5).

**TABLE 5:** Procedure-related complications and safety outcomes.

Outcome	Total *n*/*N* (%)	Comparison groups	*P*-value	Effect size
Any complication	13/121 (10.7%)	≥2 procedures: **6/30 (20.0%)** vs. single procedure: **7/91 (7.7%)**	0.059**	OR = **3.00** (95% CI: 0.92–9.77)
Pneumothorax	9/121 (7.4%)	TTNB: **6/23 (26.1%)** vs. no TTNB: **3/98 (3.1%)**	<0.001*	OR = **11.18** (95% CI: 2.55–49.04)
		FOB/TBB: 4/65 (6.2%) vs. no FOB/TBB: 5/56 (8.9%)	0.562	OR = 0.67 (95% CI: 0.17–2.62)
		EBUS: 2/47 (4.3%) vs. no EBUS: 7/74 (9.5%)	0.288	OR = 0.43 (95% CI: 0.09–2.14)
		VATS: 2/19 (10.5%) vs. no VATS: 7/102 (6.9%)	0.576	OR = 1.60 (95% CI: 0.31–8.35)
Bleeding	4/121 (3.3%)	FOB/TBB: 2/65 (3.1%) vs. no FOB/TBB: 2/56 (3.6%)	0.879	OR = 0.86 (95% CI: 0.12–6.29)
		TTNB: 1/23 (4.3%) vs. no TTNB: 3/98 (3.1%)	0.756	OR = 1.44 (95% CI: 0.14–14.50)
		EBUS: 2/47 (4.3%) vs. no EBUS: 2/74 (2.7%)	0.642	OR = 1.60 (95% CI: 0.22–11.76)
		VATS: 2/19 (10.5%) vs. no VATS: 2/102 (2.0%)	0.055**	OR = 5.88 (95% CI: 0.78–44.62)
≥2 invasive procedures	27/121 (22.3%)	Miners: **12/30 (40.0%)** vs. non-miners: **18/91 (19.8%)**	0.026*	OR = **2.70** (95% CI: 1.11–6.61)
Any complication among ≥2 procedures	6/30 (20.0%)	≥2 procedures vs. single procedure	0.059**	OR = 3.00 (95% CI: 0.92–9.77)
Procedure-related mortality	0	–	–	–

*Pearson’s chi-squared test.

**Fisher exact test. FOB/TBB, fiberoptic bronchoscopy with transbronchial biopsy; EBUS, endobronchial ultrasound; TTNB, transthoracic needle biopsy; VATS, video-assisted thoracoscopic surgery. Bold values indicate statistical significance (*P* < 0.05).

## 4 Discussion

In this retrospective cohort of pneumoconiosis patients undergoing invasive diagnostic evaluation, approximately one-fifth of all pneumoconiosis cases diagnosed during the study period had undergone at least one invasive procedure. This finding highlights the substantial diagnostic complexity of dust-related interstitial lung disease when radiological patterns overlap with malignancy, infection, or inflammatory conditions. From a public health perspective, this also suggests that a considerable proportion of patients with occupational lung disease may be exposed to potentially avoidable invasive procedures during the diagnostic process. While our cohort represents a selected population in whom pneumoconiosis was ultimately confirmed after invasive assessment, this focused design provides clinically relevant insight into diagnostic pathways and procedure-related safety specifically within confirmed occupational lung disease. This selection reflects a population enriched for diagnostic uncertainty rather than the general spectrum of pneumoconiosis. Therefore, the observed diagnostic discordance and procedural patterns should be interpreted within the context of spectrum bias and may not be generalizable to all pneumoconiosis patients. Rather than representing misclassification, this design captures diagnostic complexity in patients who underwent invasive evaluation. It should also be acknowledged that pneumoconiosis is a heterogeneous category encompassing distinct entities with differing radiological and pathological features. In our cohort, silicosis was predominant, followed by welders’ pneumoconiosis and a single case of asbestosis. Although all pneumoconioses were analyzed together to reflect the real diagnostic challenge encountered in routine practice, this heterogeneity should be considered when interpreting the findings.

Recent CT-based studies have also demonstrated that radiological patterns in pneumoconiosis may vary according to exposure duration and type, highlighting the heterogeneity of imaging findings across different occupational settings (5). Radiological severity was consistently associated with impaired pulmonary function. PMF and higher profusion categories were linked to significantly lower FVC, and profusion score correlated inversely with lung function, reinforcing prior evidence that complicated pneumoconiosis carries substantial physiological burden (6, 7). These findings underscore that radiological grading in pneumoconiosis extends beyond descriptive imaging classification and reflects clinically meaningful disease severity that may influence both symptom burden and downstream diagnostic decision-making as well as procedural escalation in routine care (6, 8, 9).

A particularly notable finding was the very high rate of diagnostic discordance (82.6%) between the leading preprocedural clinical impression and the diagnosis established through invasive evaluation. This likely reflects the substantial radiological overlap between pneumoconiosis and conditions such as malignancy, tuberculosis, and inflammatory lung diseases in routine clinical settings. In exploratory multivariable analysis, absence of comorbidity independently predicted discordance, suggesting that limited competing clinical context may broaden differential diagnosis and increase the likelihood of an initially inaccurate working diagnosis. The higher diagnostic concordance in patients with comorbidities (COPD, asthma, hypertension, diabetes mellitus, and coronary artery disease) may have several explanations. These patients are usually under regular medical follow-up, with more extensive prior imaging and documentation, allowing a more focused working diagnosis when a new pulmonary lesion is identified. Comorbidities, along with associated factors such as older age and smoking, may also increase the pretest probability of conditions such as malignancy, making the leading working diagnosis more likely to be confirmed. Importantly, this finding emphasizes the central role of structured occupational exposure assessment and multidisciplinary interpretation in patients presenting with nodular or mass-like pulmonary lesions, particularly outside specialized occupational disease centers. Failure to incorporate occupational exposure early in the diagnostic process may contribute to misdirected clinical suspicion and unnecessary invasive procedures. Rather than indicating diagnostic failure, the observed discordance illustrates the inherent complexity of evaluating suspicious pulmonary lesions in patients undergoing lesion-targeted diagnostic evaluation. In this context, diagnostic discordance may partly reflect inherent diagnostic uncertainty rather than true misclassification.

Invasive diagnostic procedures were associated with a measurable but overall modest complication burden (10.7%), with pneumothorax and bleeding as the most frequent adverse events and no procedure-related mortality. Pneumothorax clustered predominantly in patients undergoing TTNB, consistent with broader CT-guided biopsy literature where complication risk is influenced by lesion location and underlying parenchymal disease (10–12). The strong association between TTNB and pneumothorax in our cohort (OR > 11) highlights the need for careful procedural selection in patients with fibrotic lung architecture. Even when complication rates are relatively low, the cumulative impact of invasive procedures at the population level should not be overlooked, particularly when such procedures may be avoidable with improved diagnostic pathways. Although bleeding events were infrequent, a numerically higher rate following VATS suggests that cumulative procedural invasiveness may contribute to complication risk, particularly when multiple sampling approaches are employed.

Distinct occupational groups followed different diagnostic trajectories. Welders (predominantly diagnosed with welders’ pneumoconiosis) were more likely to receive an initial working diagnosis of hypersensitivity pneumonitis and more frequently underwent bronchoscopic sampling, alongside lower prevalence of PMF and mass-like lesions. This pattern suggests that exposure type and radiological phenotype jointly shape diagnostic reasoning. Dental technicians demonstrated high EBUS utilization, reflecting mediastinal involvement patterns described in mixed-dust exposures (13, 14). Of note, miners, stoneworkers, foundry workers, and dental technicians were predominantly diagnosed with silicosis, reflecting shared silica exposure despite their differing occupational settings; this common underlying entity, presenting through varied radiological phenotypes, may partly explain the divergent diagnostic trajectories observed across these groups. Miners had a significantly higher likelihood of undergoing multiple invasive procedures, potentially reflecting greater diagnostic uncertainty or more complex radiological presentations. Notably, although miners might be expected to be readily recognized as having pneumoconiosis given the classical association of mining with dust exposure, an initial working diagnosis of pneumoconiosis was frequently not made in this group. The prevalence of PMF (60.0% vs. 54.9%, *p* = 0.676) and mass-like lesions (63.3% vs. 57.1%, *p* = 0.670) did not differ significantly between miners and other occupational groups, indicating that this lack of initial recognition was not attributable to a higher burden of pseudotumoral or mass-like presentations specific to miners. Rather, mass-like and PMF-type presentations were common across the entire cohort, and in this context radiologically suspicious lesions appear to have prompted exclusion of malignancy or infection before occupational exposure was considered, regardless of occupation. This suggests that the failure to suspect pneumoconiosis initially reflected insufficient early integration of occupational history rather than an atypical radiological phenotype unique to miners. These occupation-specific diagnostic pathways highlight variability in clinical decision-making that may have implications for healthcare utilization and procedural burden across different worker populations. These occupation-specific trajectories underscore the importance of integrating detailed exposure history into radiological interpretation to reduce repeated or sequential invasiveness (15).

Collectively, our findings support a more structured, phenotype-informed diagnostic framework in pneumoconiosis patients presenting with radiologically suspicious lesions. Radiological pattern recognition (e.g., consolidation favoring bronchoscopic approaches, peripheral mass-like lesions prompting percutaneous strategies) should be systematically integrated with occupational exposure assessment and pulmonary functional evaluation. From a public health standpoint, strengthening early occupational exposure assessment and multidisciplinary evaluation may help reduce unnecessary invasive procedures, optimize resource utilization, and improve patient safety. Such an approach may improve diagnostic efficiency, reduce unnecessary invasive procedures, and optimize patient safety in occupational lung disease practice (16, 17).

This study has several strengths. To our knowledge, it is among the few studies to specifically characterize the diagnostic pathway and procedural burden experienced by pneumoconiosis patients before their occupational etiology is recognized, an aspect that has received limited attention in the existing literature. All diagnoses were established within a dedicated occupational disease center through multidisciplinary evaluation integrating exposure history, imaging, and clinical assessment, providing a high level of diagnostic confidence. The cohort was drawn from a well-defined population of 550 consecutively diagnosed pneumoconiosis patients over a 5-year period, and procedure-specific outcomes — including phenotype-driven procedure selection and complication profiles — were systematically documented. Finally, the occupation-stratified analyses provide granular, clinically applicable insight into how exposure type shapes diagnostic decision-making in routine practice.

### 4.1 Limitations

This study has several limitations. First, its retrospective single-center design limits causal inference and external generalizability. As a tertiary referral occupational disease center, our cohort may reflect a higher proportion of diagnostically complex or advanced cases compared with community-based settings.

Second, inclusion was restricted to patients who underwent invasive diagnostic procedures and were ultimately diagnosed with pneumoconiosis. This enrichment strategy enabled focused evaluation of diagnostic pathways and procedural safety within confirmed occupational lung disease but likely overrepresents diagnostically complex cases and advanced radiological phenotypes. Patients undergoing invasive evaluation in whom pneumoconiosis was excluded were not systematically recorded and were therefore not included in the analysis. Consequently, the present findings do not reflect the full spectrum of differential diagnostic work-up for nodular lung disease and may be subject to spectrum bias, reflecting a population enriched for diagnostic uncertainty rather than the general pneumoconiosis spectrum, thereby limiting generalizability. However, this design also provides targeted insight into a clinically relevant subgroup in which diagnostic uncertainty may lead to increased use of invasive procedures.

Third, event counts for diagnostic concordance and complications were limited, and multivariable models should be interpreted as exploratory. The study was not powered for definitive risk prediction modeling.

Finally, detailed quantitative exposure metrics and longitudinal outcome data were not available. The lack of quantitative exposure assessment limits causal inference and precludes formal exposure–response analysis, as exposure intensity and cumulative dose could not be systematically evaluated. Additionally, the absence of longitudinal follow-up data precluded assessment of long-term clinical outcomes. These limitations also restrict evaluation of the broader public health impact and long-term consequences of invasive diagnostic pathways in occupational lung disease. Future prospective multicenter studies incorporating standardized diagnostic algorithms and longitudinal follow-up are warranted.

## 5 Conclusion

In routine clinical practice, pneumoconiosis frequently mimics malignancy, infection, and inflammatory lung diseases, leading to invasive diagnostic evaluation before occupational etiology is recognized. Radiological phenotype and functional impairment were closely associated with procedural selection, while diagnostic discordance between initial working diagnosis and final diagnosis was common, particularly in patients without significant comorbidity. Although overall procedural safety was acceptable, pneumothorax was strongly associated with percutaneous biopsy approaches, underscoring the need for careful procedural selection in patients with fibrotic lung architecture.

From a public health perspective, these findings suggest that inadequate integration of occupational exposure assessment into early diagnostic pathways may contribute to potentially avoidable invasive procedures and increased healthcare burden.

These findings highlight the importance of early occupational exposure assessment, multidisciplinary interpretation, and phenotype-informed diagnostic pathways to improve diagnostic efficiency, reduce unnecessary invasiveness, and optimize patient safety in occupational lung disease practice.

## Data Availability

The raw data supporting the conclusions of this article will be made available by the authors, without undue reservation.
