# Supplementary material for: Diagnostic complexity and potentially avoidable invasive procedures before the recognition of pneumoconiosis
Source: Front Med (Lausanne). 2026 Jun 26;13:1842645. doi: 10.3389/fmed.2026.1842645 (PMC13351977; doi:10.3389/fmed.2026.1842645)
Supplement: Supplementary file 1 [file Data_Sheet_1.DOCX]

# STROBE Checklist – Observational Study

Title of study: Occupational Exposure–Related Diagnostic Complexity and Invasive Procedures in Pneumoconiosis: A Real-World Cohort Study

Manuscript details:

Word count: 3673 words

Pages: 17 pages

Study design: Single-center retrospective cohort study

Main Figures and Tables:

Figure 1. Decline in forced vital capacity across increasing radiological profusion categories.

Table 1. Baseline demographic, clinical, radiological, and functional characteristics of the study population.

Table 2. Association between radiological features and diagnostic procedure selection in patients with pneumoconiosis (n = 121).

Table 3. Association between radiological severity and pulmonary function parameters.

Table 4. Diagnostic concordance and predictors of diagnostic discordance.

Table 5. Procedure-related complications and safety outcomes.

Online Supplementary Material:

Supplementary Figure S1. Flow diagram of patient selection.

Supplementary Table S1. Association between occupational groups and diagnostic procedure selection in patients with pneumoconiosis (n = 121).

Supplementary Table S2. Clinical and radiological characteristics associated with welding occupation in pneumoconiosis.

Supplementary Table S3. Clinical and functional determinants of initial diagnostic impressions.

STROBE Checklist Items:

1. Title and abstract – Study design clearly indicated in abstract.

2. Background/rationale – Scientific background and justification described in Introduction.

3. Objectives – Specific objectives stated in Abstract and Introduction.

4. Study design – Key elements of design described in Methods.

5. Setting – Clinical setting, location, and study period reported.

6. Participants – Eligibility criteria and selection explained; flow diagram in Supplementary Figure S1.

7. Variables – Outcomes, exposures, predictors, confounders defined in Methods.

8. Data sources/measurement – Imaging, spirometry, occupational data collection described.

9. Bias – Potential sources of bias addressed in Discussion (limitations).

10. Study size – Sample derivation explained from 550 to 121 patients.

11. Quantitative variables – Handling and categorisation described in Statistical Analysis.

12. Statistical methods – All analyses described including regression and subgroup analyses.

13. Participants flow – Numbers at each stage shown in Supplementary Figure S1.

14. Descriptive data – Baseline characteristics reported in Table 1.

15. Outcome data – Radiological severity, procedures and complications reported in Tables 2–5.

16. Main results – Effect estimates and statistical significance reported in Results.

17. Other analyses – Subgroup analyses presented in Supplementary Tables S1–S3.

18. Key results – Summarised in Discussion and Conclusions.

19. Limitations – Addressed in Discussion.

20. Interpretation – Balanced interpretation provided.

21. Generalisability – External validity discussed.

22. Funding – No external funding declared.

23. Competing interests – Statement included in manuscript.
