# Supplementary material for: Diagnostic complexity and potentially avoidable invasive procedures before the recognition of pneumoconiosis
Source: Front Med (Lausanne). 2026 Jun 26;13:1842645. doi: 10.3389/fmed.2026.1842645 (PMC13351977; doi:10.3389/fmed.2026.1842645)
Supplement: Supplementary file 2 [file Data_Sheet_2.DOCX]

**FIGURE LEGEND**

**Supplementary Figure S1. Flow diagram of patient selection.**


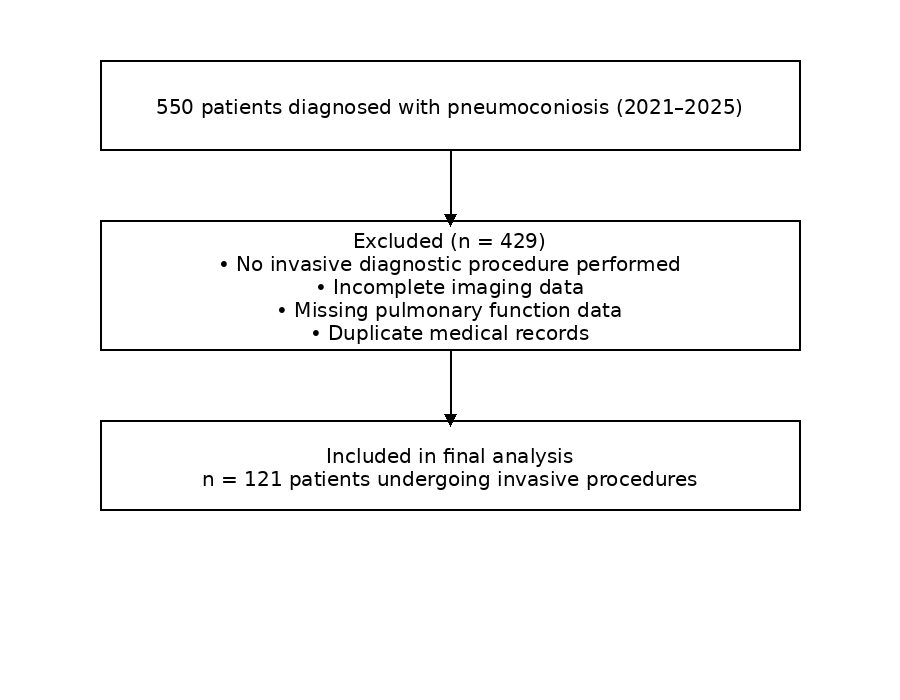


Among 550 patients diagnosed with pneumoconiosis between 2021 and 2025, 121 patients who underwent at least one invasive diagnostic procedure and had complete imaging and functional data were included in the final analysis. The remaining patients were excluded due to absence of invasive procedures, incomplete imaging, missing functional data, or duplicate records

**SUPPLEMENTARY TABLES**

**Supplementary Table S1.** **Association between occupational groups and diagnostic procedure selection in patients with pneumoconiosis (n = 121).**

| **Occupational Groups** | **Diagnostic Procedure** | **Feature (+) n/N (%)** | **Feature (-) n/N (%)** | **p value** |
| --- | --- | --- | --- | --- |
| **Miner (n=30)** | FOB/TBB | 13/30 (43.3%) | 52/91 (57.1%) | 0.188 |
|  | TTNB | 9/30 (30.0%) | 14/91 (15.4%) | 0.077 |
|  | EBUS | 16/30 (53.3%) | 31/91 (34.1%) | 0.084 |
|  | VATS | 4/30 (13.3%) | 15/91 (16.5%) | 0.681 |
|  | ≥2 invasive procedures | 12/30 (40.0%) | 18/91 (19.8%) | **0.026*** |
| **Welder (n=25)** | FOB/TBB | **23/25 (92.0%)** | 42/96 (43.8%) | **<0.001*** |
|  | TTNB | **1/25 (4.0%)** | 22/96 (22.9%) | **0.042**** |
|  | EBUS | **2/25 (8.0%)** | 45/96 (46.9%) | **<0.001*** |
|  | VATS | 2/25 (8.0%) | 17/96 (17.7%) | 0.357 |
|  | ≥2 invasive procedures | 3/25 (12.0%) | 27/96 (28.1%) | 0.096 |
| **Foundry worker (n=16)** | FOB/TBB | 9/16 (56.2%) | 56/105 (53.3%) | 0.827 |
|  | TTNB | 3/16 (18.8%) | 20/105 (19.0%) | 0.977 |
|  | EBUS | 7/16 (43.8%) | 40/105 (38.1%) | 0.666 |
|  | VATS | 0/16 (0.0%) | 19/105 (18.1%) | 0.073 |
|  | ≥2 invasive procedures | 3/16 (18.8%) | 27/105 (25.7%) | 0.548 |
| **Dental technician (n=14)** | FOB/TBB | **3/14 (21.4%)** | 62/107 (57.9%) | **0.010*** |
|  | TTNB | 2/14 (14.3%) | 21/107 (19.6%) | 0.632 |
|  | EBUS | **10/14 (71.4%)** | 37/107 (34.6%) | **0.008*** |
|  | VATS | 1/14 (7.1%) | 18/107 (16.8%) | 0.349 |
|  | ≥2 invasive procedures | 2/14 (14.3%) | 28/107 (26.2%) | 0.333 |
| **Stonework (n=11)** | FOB/TBB | 5/11 (45.5%) | 60/110 (54.5%) | 0.564 |
|  | TTNB | 4/11 (36.4%) | 19/110 (17.3%) | 0.124 |
|  | EBUS | 4/11 (36.4%) | 43/110 (39.1%) | 0.860 |
|  | VATS | 3/11 (27.3%) | 16/110 (14.5%) | 0.269 |
|  | ≥2 invasive procedures | 4/11 (36.4%) | 26/110 (23.6%) | 0.351 |

**Pearson's chi-squared test****, ******Fisher exact test*

***Note:*** *Percentages indicate the proportion of patients undergoing two or more invasive diagnostic procedures during the diagnostic work-up.*

**Abbreviations:** FOB/TBB, fiberoptic bronchoscopy with transbronchial biopsy; EBUS, endobronchial ultrasound; TTNB, transthoracic needle biopsy; VATS, video-assisted thoracoscopic surgery

**Supplementary Table S2.** **Clinical and radiological characteristics associated with welding occupation in pneumoconiosis.**

| **Variable** | **Welder**  (**n = 25) n, %** | **Non-welder**  **(n = 96) n, %** | **p value** |
| --- | --- | --- | --- |
| ***Clinical characteristics*** | | | |
| **Initial HP impression** | 11 (44.0%) | 5 (5.2%) | **<0.001*** |
| ***Radiological features*** | | | |
| **PMF** | 3 (12.0%) | 65 (67.7%) | **<0.001*** |
| **Pulmonary mass** | 5 (20.0%) | 66 (68.8%) | **<0.001*** |
| **Atypical lesion location** | 1 (4.0%) | 10 (10.4%) | 0.456 |
| **Pleural effusion** | 0 (0.0%) | 11 (11.5%) | 0.118 |
| **Consolidation** | 19 (76.0%) | 46 (47.9%) | **0.012*** |
| **Cavity** | 0 (0.0%) | 4 (4.2%) | 0.580 |
| **LAP** | 15 (60.0%) | 84 (87.5%) | **0.003*** |

**Pearson's chi-squared test*

**Abbreviations:** PMF, progressive massive fibrosis; HP, hypersensitivity pneumonitis; LAP, lymphadenopathy.

**Supplementary Table S3.** **Patient characteristics according to initial clinical impression prior to invasive diagnostic procedures**

| **Initial Diagnostic Impression** | **Variable** | **Diagnosis (+)** | **Diagnosis (−)** | **p value** |
| --- | --- | --- | --- | --- |
| **Infection**  **(n = 5)** | Age (years), Mean ± SD | 57.2 ± 13.7 | 55.0 ± 12.2 | 0.701 |
|  | FVC (%pred), Mean ± SD | 54.0 ± 23.0 | 82.2 ± 23.8 | **0.022*** |
|  | Exposure duration, Mean ± SD | 17.8 ± 14.4 | 20.7 ± 10.4 | 0.545 |
| **Malignancy**  **(n = 60)** | Age (years), Mean ± SD | 58.7 ± 12.8 | 51.6 ± 10.6 | **0.001*** |
|  | FVC (%pred), Mean ± SD | 78.1 ± 23.0 | 84.4 ± 25.2 | 0.158 |
|  | Exposure duration, Mean ± SD | 21.8 ± 11.1 | 19.4 ± 9.9 | 0.214 |
| **Tuberculosis**  **(n = 22)** | Age, Mean ± SD | 54.5 ± 11.9 | 55.3 ± 12.3 | 0.775 |
|  | FVC, Mean ± SD | 74.1 ± 21.4 | 82.8 ± 24.6 | 0.125 |
|  | Exposure duration, Mean ± SD | 20.5 ± 10.7 | 20.6 ± 10.6 | 0.957 |
| **Hypersensitivity pneumonitis**  **(n = 16)** | Age (years), Mean ± SD | 48.7 ± 9.1 | 56.1 ± 12.4 | **0.023*** |
|  | FVC (%pred), Mean ± SD | 93.3 ± 24.0 | 79.3 ± 23.8 | **0.031*** |
|  | Exposure duration, Mean ± SD | 20.8 ± 79.1 | 20.6 ± 10.9 | 0.935 |
| **Sarcoidosis**  **(n = 15)** | Age (years), Mean ± SD | 49.5 ± 8.5 | 55.9 ± 12.5 | 0.057 |
|  | FVC (%pred), Mean ± SD | 94.1 ± 21.5 | 79.4 ± 24.1 | **0.027*** |
|  | Exposure duration, Mean ± SD | 18.1 ± 10.5 | 21.0 ± 10.6 | 0.333 |
| **Interstitial lung disease**  **(n = 3)** | Age (years), Mean ± SD | 48.0 ± 8.7 | 55.3 ± 12.3 | 0.307 |
|  | FVC (%pred), Mean ± SD | 114.5 ± 0.7 | 80.6 ± 24.0 | **0.050**** |
|  | Exposure duration, Mean ± SD | 13.3 ± 2.1 | 20.8 ± 10.6 | 0.228 |

**t-test; **Borderline significance; exploratory*

**Abbreviations:** FVC, forced vital capacity; SD, standard deviation.

***Note:*** *Diagnosis (+) indicates patients with the specified initial clinical impression documented prior to invasive diagnostic procedures; Diagnosis (−) indicates all remaining patients. Numbers of patients for each initial diagnosis are provided in the row headers. Subgroup sizes for infection, interstitial lung disease and hypersensitivity pneumonitis were small; therefore, these analyses should be interpreted as exploratory.*
